# Long-term outcome of two-stage revision surgery after hip and knee prosthetic joint infections: an observational study

**DOI:** 10.5194/jbji-6-379-2021

**Published:** 2021-10-20

**Authors:** Yorrick P. Bourgonjen, J. Fred F. Hooning van Duyvenbode, Bruce van Dijk, F. Ruben H. A. Nurmohamed, Ewout S. Veltman, H. Charles Vogely, Bart C. H. van der Wal

**Affiliations:** Department of Orthopedics, University Medical Center Utrecht, Utrecht, the Netherlands

## Abstract

**Introduction**: Two-stage revision surgery is the most frequently performed procedure in patients with a chronic periprosthetic joint infection (PJI). The infection eradication rates in the current literature differ between 54 % and 100 %, which could be attributed to different treatment strategies. The aim of this study was to retrospectively evaluate the infection eradication rate in patients with chronic PJI treated with two-stage revision surgery of the hip or knee in primary and re-revision cases. **Methods**: All patients treated with a two-stage revision for chronic PJI between 2005 and 2011 were analysed. Patient and infection characteristics were retrieved. Primary outcome was successful infection eradication at last follow-up. Successful eradication is specified as no need for subsequent revision surgery or suppressive antibiotic treatment. **Results**: Forty-seven patients were treated with a two-stage revision. Infection eradication was achieved in 36 out of 47 cases. Thirty-eight patients had positive cultures: 35 monomicrobial infections and 3 polymicrobial infections. Nine cases of culture-negative infections were identified. Accompanying eradication rates were 26 out of 35 cases, 2 out of 3 cases, and 8 out of 9 cases respectively. Mean follow-up was 128 (27–186) months. For hip and knee revisions the eradication rates were 22 out of 31 cases and 14 out of 16 cases respectively. After primary arthroplasty the infection was eradicated in 29 out of 38 cases and after re-revision in 7 out of 9 cases. **Conclusion**: In this study, the infection eradication rate for two-stage revision surgery after PJI of the hip and knee in primary and re-revision cases was 77 %. No statistically significant patient, infection and micro-organism characteristics were found which influence the infection eradication rates at long-term follow-up of 128 (27–186) months.

## 1 Introduction

Periprosthetic joint infection (PJI) is one of the most feared complications in joint replacement surgery. It is often associated with pain, hospitalization, (multiple) surgical intervention(s), (irreversible) loss of function, and increased mortality (Li et al., 2018). Moreover, PJI leads to a significant burden for the hospital and healthcare systems at high and rising costs (Kurtz et al., 2012). With the increasing number of joint arthroplasties, the number of PJIs is rising. The increasing number of arthroplasties combined with an increased expected lifespan puts more patients at risk of late PJI (Huotari et al., 2015). Previous studies show that PJI incidences range from 1 % to 3 % after primary total knee arthroplasty (TKA) (Wang et al., 2018; Springer et al., 2017) and from 1 % to 2 % for primary total hip arthroplasty (THA) (Springer et al., 2017). In the Netherlands, PJI is the leading cause of revision surgery within the first year after primary hip and knee arthroplasty (De Reus et al., 2019).

Two-stage revision surgery is the most frequently performed procedure in patients with a chronic PJI. Previous studies on two-stage revision show very high infection eradication rates, including some even as high as 90 %–100 % (Silvestre et al., 2013; Klouche et al., 2012; Hsieh et al., 2004). However, the results in the literature differ greatly, between 54 % and 100 % (Pangaud et al., 2019; Romano et al., 2012).

PJI can be difficult to treat due to the bacteria forming a biofilm consisting of a structured aggregation of microbial cells in a matrix which adhere to a prosthesis. It is a multifactorial problem with the existence of slow-/non-growing bacteria within the biofilm as the main contributing factor (Gbejuade et al., 2015). This biofilm hinders the immune system in adequately responding to the infection as well as making it unresponsive to most antibiotics. On top of this physical barrier, there are many risk factors associated with PJI which can limit the treatment options. These risk factors include the patient's immune status, time after primary arthroplasty, and causative micro-organism.

The aim of this study was to retrospectively evaluate the infection eradication rate in patients with chronic PJI treated with two-stage revision surgery of the hip or knee in primary and re-revision cases.

## 2 Methods and characteristics

All patients who were treated in our hospital for chronic PJI with a two-stage revision surgery between 1 January 2005 and 1 January 2011 were analysed. Patients' medical records were reviewed and collected in a database. The diagnosis of PJI was retrospectively reconfirmed according to the MSIS 2014 criteria (Parvizi and Gehrke, 2014).

Patient and infection characteristics, treatment outcome, and complications were retrieved. Primary outcome was stated as successful PJI eradication after a two-stage revision during the years of follow-up, which had to be a minimum of 2 years. If no reimplantation is performed and the patient is left with a definite Girdlestone situation, the infection could still be eradicated. An infection was considered successfully eradicated in case of the absence of clinical signs, such as pain, swelling, and erythema, radiological signs, such as prosthesis loosening, and laboratory signs, such as C-reactive protein (CRP) <10. The treatment was identified as failed when subsequent surgery was needed for persistent infection (with exclusion of a single DAIR within 3 months after the second stage) and the need for suppressive antibiotics. This study is in accordance with the STROBE guidelines (von Elm et al., 2007).

### 2.1 Patient's characteristics

Demographic and patient's characteristics, including age, sex, body mass index (BMI), American Society of Anesthesiologists (ASA) score, previous treatment on the infected site, and co-morbidities, were retrieved from the patients' medical records. Previous treatment prior to the surgery of the infected site could consist of a single debridement, antibiotics, and implant retention (DAIR), multiple DAIRs, antibiotic treatment, revision surgery (one-stage or two-stage), or a combination of treatments.

### 2.2 Infection characteristics

To characterize the infection, multiple variables were included. These characteristics were infection location, soft tissue involvement, and the type of infection. Furthermore, patients were scored according to the infection classification system by Zimmerli et al. (2004). Chronic PJI was defined as persisting infection more than 3 months after implantation. The time after primary arthroplasty and the interval between both stages were calculated and recorded in the database.

### 2.3 The two-stage procedure

A two-stage revision consists of two surgical interventions. In the first procedure, the infected prosthetic joint is extracted along with all the tissue suspected of being infected. A minimum of five fluid and/or tissue samples are acquired for culture growth, which are cultured for 2 weeks. The surgical site is extensively debrided and irrigated. Afterwards an antibiotic agent could be implanted, either gentamicin beads or an antibiotic-loaded spacer. Treatment options for the knee include an articulating or static antibiotic-loaded cement spacer. The decision to use an articulating or static spacer depends on multiple factors, with the soft tissue quality being the main contributing factor. Treatment options for the hip include a prefabricated antibiotic-loaded cement spacer or a Girdlestone situation (with or without local gentamicin beads). A Girdlestone situation and gentamicin beads are practised in the interval between both stages when there is poor bone and/or soft tissue quality, a source of infection communicating with the joint region, and/or a femur fracture during the first stage.

**Table 1:** Baseline characteristics.

	Total	Successful eradication	Failed eradication
	n=47	n=36	n=11
Patient characteristics			
Age	68 (34–84)	69 (34–84)	67 (44–79)
Sex (M/F)${}^{a}$	26/21	20/16	6/5
BMI${}^{b}$	28.9 (20.5–52.0)	27.9 (20.5–35.1)	31.5 (20.9–52.0)
ASA${}^{c}$ score 1/2/3/4	8/30/8/1	6/27/3/0	2/3/5/1
Previous infection treatment	27	21	6
Time after primary arthroplasty (months)	64 (3–243)	57 (4–200)	86 (3–243)
Re-revision	9	7	2
Risk factors			
Diabetes mellitus	7	5	2
Cardiovascular disease	22	16	6
Smoking	8	3	5
Alcohol abuse	7	4	3
Infection characteristics			
Treatment location			
Hip	31	22	9
Knee	16	14	2
Zimmerli infection type			
<3 months	0	0	0
3–24 months	13	11	2
>24 months	34	25	9
Soft tissue involvement			
Abscess or fistula	19	14	5

${}^{a}$ M/F: male/female; ${}^{b}$ body mass index; ${}^{c}$ American Society of Anesthesiologists.

During the interval period, after culture results became final, a multidisciplinary meeting with the infectiologist and medical microbiologist determined at what moment the second stage of the treatment could be planned. In case of negative cultures the second-stage procedure was planned with a 2- to 4-week interval. In case of positive cultures the antibiotic treatment was prolonged and adjusted to the sensitivity pattern of the causative pathogen. Preferably antibiotics were ceased at least 2 weeks preoperatively to create an antibiotics-free period. Patients were treated with oral antibiotics whenever possible, according to micro-organism sensitivity, during the two-stage interval, and outpatient clinic visits were planned to assess the infection status. Reimplantation of the prosthesis was planned dependent on multiple factors, including the patient's bone quality, soft tissue quality, and wound healing, infection parameters before and after the first stage, such as CRP and erythrocyte sedimentation rate (ESR), and micro-organism sensitivity.

During the second-stage procedure the local antibiotic carrier is extracted, and a minimum of five fluid or tissue samples were taken for culture. The joint is extensively debrided followed by reimplantation of a hip or knee prosthesis.

### 2.4 Microbiology characteristics and antibiotic treatment

All patients received systemic antibiotics in combination with the surgical and local antibiotic treatment. It started with intravenous antibiotic treatment, for a minimum of 2 weeks, and was hereafter switched to oral treatment if possible. In the case of an unknown causative micro-organism, the antibiotic of first choice would be cefazolin. The resistance pattern of the cultures taken during the first stage determined the choice of antibiotics. The antibiotic treatment is continued until the cultures taken during the second stage are conclusive. If these returned negative, 6 weeks of antibiotic treatment were indicated. If these returned positive for micro-organisms, 12 weeks of antibiotic treatment were indicated.

During the first procedure and second procedure multiple samples are taken for microbial culture and assessed by a microbiologist. If two or more positive cultures identified the same causative organism, an infection was considered to be proven. The infection was classified as polymicrobial when two or more micro-organisms were identified.

### 2.5 Data and statistical analysis

Data are presented using either a mean or median. Multiple patient and infection characteristics were analysed for their influence on the infection eradication rate using Firth's logistic regression. For all statistics a p value ≤0.05 was considered significant. Data management and analysis were performed using the Statistical Package for the Social Sciences (IBM Corp. Released 2017. IBM SPSS Statistics for Windows, version 25.0. Armonk, NY: IBM Corp.) and using the statistical software SAS^®^ Studio University Edition (SAS Institute. Released 2018. Version 3.8 (Basic Edition), SAS Institute Inc., Cary, NC, USA).

**Figure 1:**
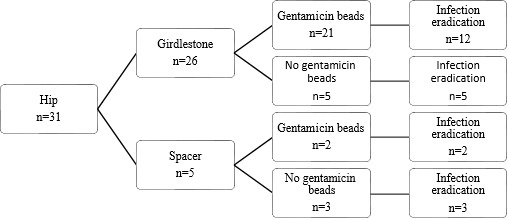
Flow diagram of treatment strategy and infection eradication for patients treated for PJI of the hip.

**Figure 2:**
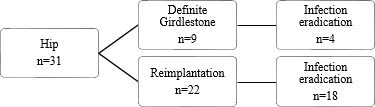
Flow diagram of reimplantation and infection eradication for patients treated for PJI of the hip.

**Figure 3:**
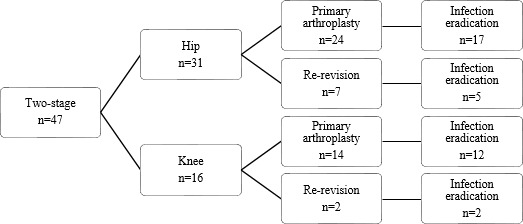
Flow diagram of infection eradication for patients treated for PJI after primary arthroplasty compared with re-revision.

## 3 Results

During the study period 50 patients were treated with two stages for chronic PJI of the hip or knee. Forty-seven patients met the inclusion criteria, while three patients were excluded for not meeting the follow-up criteria. Baseline patient and infection characteristics are shown in Table 1. Successful eradication was achieved in 36 out of the 47 cases. Out of the 11 failed cases, six patients had a chronic infection, which required lifelong antibiotic suppression therapy, three patients had a definite Girdlestone situation with a chronic fistula, and two patients had a re-infection after respectively 9 months and 6 years with the same micro-organism species as the first infection, which were successfully treated with a DAIR followed by 3 months of antibiotic treatment. Thirty-eight patients received a prosthesis reimplantation. In four patients the infection was eradicated, but a reimplantation of the prosthesis was not attempted, and a definite Girdlestone situation was accepted. No re-extractions of the revised prostheses were required. The mean follow-up was 128 (27–186) months.

### 3.1 Hip revision surgery

Treatment strategies and results can be found in Figs. 1, 2, and 3. Twenty-two patients had a prosthesis reimplantation, while in nine patients a definite Girdlestone situation was accepted. Out of those nine patients, four had successful infection eradication. Four patients with a failed treatment had a persistent infection, with a fistula and/or abscess, requiring suppressive antibiotic treatment. The other patient in the failed group with a definite Girdlestone situation had a chronically infected femoral artery prosthesis, which connected with the hip joint, requiring lifelong antibiotic suppression therapy. Out of the successfully eradicated Girdlestone patients, three patients did not wish for prosthesis reimplantation, and one patient was unfit for surgery. When comparing revision after primary arthroplasty and re-revisions, it shows that the infection was successfully eradicated in 17 out of 24 cases after primary arthroplasty and 5 out of 7 cases after re-revision.

### 3.2 Knee revision surgery

**Figure 4:**
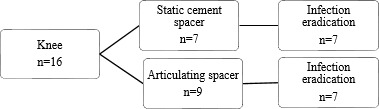
Flow diagram of treatment strategy and infection eradication for patients treated for PJI of the knee.

Treatment strategies and results can be found in Figs. 3 and 4. Out of the 16 treated knees, 15 received a spacer, and one patient received a combination of a spacer and gentamicin beads during treatment. Out of the two failed cases, one patient had a reinfection with the same micro-organism after 9 months, which was successfully treated with a DAIR followed by 3 months of antibiotics. The other patient in the failed group had a persistent infection, requiring lifelong suppressive antibiotic treatment. When comparing revision after primary arthroplasty and re-revisions, it shows that the infection was successfully eradicated in 12 out of 14 cases after primary arthroplasty and all 2 cases after re-revision.

### 3.3 Microbiology findings

The median number of cultures was 7 (5–12), with a median of 4 (0–10) positive cultures (Table 2).

Thirty-five patients had a monomicrobial infection, while three patients had a polymicrobial infection. The most commonly found micro-organism was the *coagulase-negative Staphylococcus* (CoNS), which was found in 15 patients and in combination with other micro-organisms in 2 patients. *Staphylococcus aureus* was found in five cases, including one polymicrobial case. No cases of methicillin-resistant *Staphylococcus aureus* were found. A total of 16 different micro-organisms were detected.

**Table 2:** Microbiology findings and eradication rate according to causative pathogen.

Micro-organism	Total	Successful
	(n)	eradication (n)
*CoNS*${}^{a}$	15	11
*S.*${}^{b}$ *aureus*	4	3
*Enterococci*${}^{c}$	4	1
*Enterobacterales*${}^{d}$	4	4
*Candida albicans*	3	2
*Pseudomonas aeruginosa*	2	2
*Proteus mirabilis*	1	1
*S.*${}^{b}$ *epidermis*	1	1
*Streptococcus agalactiae (B)*	1	0
Polymicrobial	3	2
Culture negative	9	8

${}^{a}$ *Coagulase-negative Staphylococcus*; ${}^{b}$ *Staphylococcus*; ${}^{c}$ *Enterococcus faecalis* (n=1), *Enterococcus faecium* (n=1), *Enterococcus casseliflavus* (n=1), Amoxicillin-resistant *Enterococcus faecium* (n=1); ${}^{d}$ *Escherichia coli* (n=1), *Enterobacter cloacae complex* (n=1), *Serratia marcescens* (n=1).

### 3.4 Risk factors

Multiple variables were analysed to explore whether they had a significant influence on the outcome of two-stage revision surgery. These variables included sex, age, BMI, time after primary arthroplasty, previous treatment, primary or revision prosthesis, infected joint, ASA classification, Zimmerli classification, and soft tissue involvement. Using Firth's logistic regression, no variables were found to have a significant influence on the outcome (Table 3).

**Table 3:** Firth's logistic regression.

Variable	PE	SE	Wald	Pr > ChiSq	OR	95 % Wald CI	
Intercept	-0.8146	0.7255	1.2607	0.2615			
Sex							
Female	Reference						
Male	-0.1073	0.4007	0.0718	0.7888	0.807	0.168	3.881
Previous treatment							
Yes	Reference						
No	0.0319	0.4341	0.0054	0.9413	1.066	0.194	5.845
Prosthesis type							
Revision	Reference						
Primary	0.00497	0.4341	0.0001	0.9932	1.010	0.103	9.925
Infection location							
Knee	Reference						
Hip	0.4090	0.4596	0.7918	0.3736	2.266	0.374	13.731
Cultures							
Positive	Reference						
Negative	-0.0601	0.5462	0.0121	0.9124	0.887	0.104	7.546
ASA classification							
>3	Reference						
<3	-0.7660	0.4232	3.3629	0.0667	0.212	0.040	1.113
Soft tissue involvement							
Negative	Reference						
Positive	-0.4528	0.4887	0.8583	0.3542	0.914	0.175	4.782
Zimmerli infection type							
>24 months	Reference						
3–24 months	-0.4528	0.4887	0.8583	0.3542	0.404	0.060	2.746
BMI							
>30	Reference						
<30	-0.2679	0.4548	0.3470	0.5558	0.585	0.098	3.480
Age	0.00717	0.0561	0.0163	0.8983	1.007	0.902	1.124
Time after primary arthroplasty (months)	-0.00117	0.00191	0.3734	0.5412	0.999	0.995	1.003

PE: point estimate, SE: standard error, Wald: Wald test, OR: odds ratio, 95 % Wald CI: 95 % Wald confidence interval, Pr > ChiSq: all predictors equal to zero.

## 4 Discussion

In this study, the infection eradication rate of PJI-related two-stage revision surgery for chronic PJI in our hospital was explored. Infection eradication was achieved in 77 % (36 out of 47) of the cases, with a mean follow-up period of 128 (27–186) months. The majority of studies on the infection eradication rate of two-stage revision surgery show a success rate of more than 80 % (Hofmann et al., 2005; Durbkhakula et al., 2004; Volin et al., 2004; Sherrell et al., 2011); however, according to Gomez et al. (2015), in most of the studies there is no clear definition of what is considered a successful PJI treatment. This could lead to an overestimation of the success rate as well as make it difficult to compare study results due to heterogeneity in treatment. Furthermore, many studies exclude patients who did not receive prosthesis reimplantation. This could lead to an overestimation of the success rate, as only patients in a condition well enough to receive reimplantation are included. In our study, 19 % (n=9) of the patients did not receive reimplantation. This is in line with other studies on this subject (Durbkhakula et al., 2004; de Carvalho et al., 2013). It is a relatively large group of patients who cannot be overlooked, as they will have a significant influence on the infection eradication rate. Including these patients will give a more realistic outcome.

A re-revision is often considered a more complex procedure and is associated with a lower success rate. When comparing the primary revisions and re-revisions, we found that when a two-stage revision is performed after a primary arthroplasty, a successful eradication rate of 76 % (29 out of 38 cases) is achieved compared to 78 % (7 out of 9 cases) for re-revision. Hirakawa et al. (1998) described higher success percentages for patients receiving a revision after primary knee arthroplasty when compared to a re-revision, 92 % vs. 41 % respectively (in 55 cases). According to Pagnano et al. (1997), in patients receiving a re-revision after hip arthroplasty, only 27 % remained infection-free after an average follow-up of 5 years (in 34 cases). The literature on this subject is scarce and mostly with small sample sizes, making it difficult to draw conclusions.

When comparing local antibiotic agents, it shows that when a spacer is used in the hip, the infection eradication rate seems higher than when a Girdlestone situation is created (5 out of 5 cases vs. 17 out of 26 cases). A reason for this difference could be that a spacer was only used when a patient had adequate soft tissues around the spacer, while patients who had soft tissue involvement were more likely to receive a Girdlestone situation (in combination with gentamicin beads). Thus, the more severe infections are more likely to be in this group. However, our population size is too small to draw definite conclusions. According to Hsieh et al. (2004) and Marczak et al. (2017), no differences between patients treated with a spacer and patients treated with prosthesis extraction and gentamicin beads was found. Hsieh et al. (2004) report that patients receiving a spacer were more likely to have better soft tissue condition, which is in line with our findings. Marczak et al. (2017) did not mention soft tissue involvement. In current treatment strategies, spacers are considered the gold standard, and gentamicin bead usage is rare.

The most commonly found micro-organism in this study was *CoNS* with 45 %: this is in line with Bejon et al. (2010). Trampuz et al. (2007) found *Staphylococcus aureus* to be the most common micro-organism, as well as Tsai et al. (2015), while our study found an occurrence rate of only 14 % for *S. aureus*. Culture-negative PJI was found in nine (19 %) cases, out of which one may be false negative due to the use of preoperative antibiotics. Bejon et al. (2010) reported high culture-negative rates of 41 %, while a study according to Berbari et al. (2018) reported a culture-negative rate of only 7 %. However, both studies did not mention the use of preoperative antibiotics. Tsai et al. (2015) reported a culture-negative rate of 19 %, often after the use of preoperative antibiotics, but did not mention the exact number of cases.

In the case of a culture-negative PJI, no personalized targeted antibiotic therapy can be administered. This could be an additional risk of failed treatment. The diagnosis of culture-negative PJI is challenging. In current microbial culture strategies, polymerase chain reaction (PCR) proves to be a valuable additional tool for detecting micro-organisms. Multiple studies on PCR in synovial fluid show a sensitivity of 80 %–90 % (Melendez et al., 2014; Bereza et al., 2013). PCR was not part of the treatment protocol in our hospital during the study period. In our study, no significant difference between the infection eradication rate of culture-positive PJI and culture-negative PJI was seen. Conflicting results are reported about culture-negative PJI. Mortazavi et al. (2011) reported a 4 times higher risk of treatment failure in the case of culture-negative PJI, while Choi et al. (2013) reported a higher success rate for culture-negative PJI. These differences in outcome may be due to heterogeneity in treatment strategies.

This study has several limitations. A weak point of this study was reflected by the retrospective design. The number of patients included in this study was relatively low, which was caused by the scarcity of PJI cases requiring two-stage revision surgery. Moreover, no data about functional outcomes were available for analysis. The choice of treatment was reflective of the inclusion period. Because gentamicin beads are now rarely used in the treatment of PJI, the generalizability of the results for future patients may be affected.

This study provides a comprehensive overview of a frequently used treatment strategy for chronic PJI of the hip and knee. The mean follow-up of over 10 years provides a good perspective of the long-term outcome of two-stage revision surgery. Moreover, not excluding patients who did not receive reimplantation ensures a realistic infection eradication rate compared to other studies which exclude these patients.

Our results show that the infection eradication rate between two stages after primary arthroplasty and a re-revision is comparable. It is obvious that we recommend a prospective multi-centre study with a larger population evaluating the outcome of currently used two-stage treatment strategies for patients with PJI of the hip or knee. Furthermore, researchers should seek to combine retrospective data from multiple centres on the subject to increase the number of included patients and therefore provide more power.

## Data Availability

The raw data were generated at the University Medical Center Utrecht, Utrecht, the Netherlands. The data of this study are available from the co-author Bart C. H. van der Wal (b.c.h.vanderwal@umcutrecht.nl) upon request.

## Code availability

The data of this study are available in the SPSS information file.
